# Psychophysiological Stress Reactivity in Monozygotic Twins with and without Takotsubo Syndrome

**DOI:** 10.3390/biomedicines10102571

**Published:** 2022-10-14

**Authors:** Mary Princip, Claudia Zuccarella-Hackl, Rebecca E. Langraf-Meister, Aju Pazhenkottil, Victoria L. Cammann, Christian Templin, Jelena-Rima Ghadri, Roland von Känel

**Affiliations:** 1 Department of Consultation-Liaison Psychiatry and Psychosomatic Medicine, University Hospital Zurich, University of Zurich, 8091 Zurich, Switzerland; 2 Clienia Schlössli AG, Psychiatric Hospital, Schlösslistrasse 8, 8618 Oetwil Am See, Switzerland; 3 Department of Cardiology, University Hospital Zurich, University of Zurich, 8091 Zurich, Switzerland

**Keywords:** takotsubo syndrome, monozygotic twins, case report, stress reactivity, blood pressure, heart rate variability, trier social stress test

## Abstract

Objective: Takotsubo syndrome (TTS) is characterized by transient left ventricular dysfunction, often elevated myocardial enzymes, and electrocardiographic changes. Previous studies suggested that an overstimulation of the sympathetic nervous system might cause TTS. However, the pathogenesis of TTS is largely unknown. Therefore, we investigated physiological stress reactivity with a standardized stress test in monozygotic twin sisters, only one of whom had experienced TTS. Methods: The 60-year-old Caucasian monozygotic twins, one with and one without a previous episode of TTS, were recruited in the Department of Cardiology at the University Hospital Zurich, Switzerland. We applied the Trier Social Stress Test (TSST) to investigate stress reactivity six weeks after the TTS. Hemodynamic measures (heart rate (HR), blood pressure (BP)), heart rate variability (HRV), plasma norepinephrine and epinephrine and salivary cortisol levels were collected immediately before and after the TSST, and 15, 45, and 90 min after TSST. The monozygotic twins differed in their hemodynamic stress response with the TTS twin showing blunted HR and BP reactivity and vagal withdrawal beyond the acute phase of stress. In contrast, the TTS twin showed a higher catecholamine and cortisol stress response with a steady increase in norepinephrine during the recovery period from stress compared to her non-TTS twin sister. Conclusion: Large studies applying a case-control design are needed to confirm blunted hemodynamic reactivity, increased catecholamine reactivity, vagal withdrawal, and increased cortisol reactivity to stress in TTS. This may advance the knowledge of psychophysiological mechanisms in TTS.

## 1. Introduction

Takotsubo Syndrome (TTS), named after an octopus trap, was first described in 1990 in Japan [1]. Symptoms of TTS are similar to those of an acute coronary syndrome and include acute chest pain, dyspnea, ECG abnormalities and elevated cardiac biomarkers [2]. Up to 90% of TTS patients are postmenopausal women [3]. A hallmark of TTS is its association with a previous stressful event. About two-thirds of TTS cases occur after an extraordinary emotional or physical stressor, while about one-third of TTS occur in the absence of an evident stressor [4].

The underlying mechanisms involved in the pathophysiology of TTS are still largely unknown. There is intriguing research suggesting overstimulation of the autonomic nervous system and alterations within the brain-heart-axis. Specifically, elevated catecholamine levels have been found at admission for TTS. Catecholamine levels are two to three times higher in patients with TTS than in patients with acute myocardial infarction [5].

Based on this literature, it has been argued that TTS is caused by an acute release of stress hormones and occurs primarily in subjects with increased vulnerability of both the coronary microcirculation and cardiac myocytes to catecholamines [6]. Supporting this, increased reactivity of the sympathetic branch of the autonomic nervous system was found in TTS patients compared to healthy controls, with the former showing increased catecholamine levels during both mental stress and physical exercise [7]. In contrast, no evidence was found in that study for a dysregulated hypothalamic-pituitary-adrenal axis (HPA-axis) or hemodynamic responses, including blood pressure (BP) and heart rate (HR) [7]. Likewise, another study found increased epinephrine and norepinephrine reactivity, and increased vasoconstriction, although similar BP and HR reactivity in response to acute mental stress in women with a prior episode of TTS compared to a healthy post-menopausal control group [8]. Baseline levels of hemodynamic measures and catecholamines were not different between groups [8]. A third study found no alterations in myocardial function, salivary cortisol or heart rate variability (HRV) responses between TTS and healthy controls at baseline and during mental stress [9]. Thus, there is still a scant literature on whether and how altered stress responses are involved in TTS patients. To further elucidate this question, we investigated hemodynamic, autonomic and HPA-axis (i.e., cortisol) stress reactivity in monozygotic twin sisters 6 weeks after one of them had experienced a TTS episode. To our knowledge, this is the first case report of a monozygotic twin pair who underwent a standardized psychosocial stress test. The results obtained might help to better understand pathophysiological mechanisms involved in TTS in addition to a genetic contribution. 

## 2. Material and Methods

### 2.1. Study Participants

The 60-year-old Caucasian monozygotic twins, one with and one without a previous episode of TTS, were recruited in the Department of Cardiology at the University Hospital Zurich, Switzerland. The psychosocial stress test was conducted on 5 February 2020 at the University Hospital Zurich. Both women gave their written informed consent to participate in the study. No financial compensation was paid. 

### 2.2. Study Procedure and Psychosocial Stress Protocol

Upon arrival at the stress laboratory, a venous catheter was inserted, followed by an initial blood sampling. While the non-TTS twin was placed in a waiting room, the TTS twin was escorted to the examination room. Subsequently, the TTS twin received a standardized breakfast (1 bread roll, 1 apple, water) followed by the installation and start of the hemodynamic monitoring system Finapres^®^ Nova. Thereafter, the TSST was applied, a widely used procedure to induce psychosocial stress under laboratory conditions [10,11]. Following a short introduction, the TTS twin was given 3 min to prepare for the test. Then, she underwent a 5-min mock job interview, and a 5-min mental arithmetic task in front of two judges (trained staff) and a video camera. Blood and saliva samples were collected immediately before and after TSST, as well as 15, 45, and 90 min after TSST. After that, the TTS twin was allowed to rest in the waiting room and the same procedure was conducted with the non-TTS twin. Both participants were debriefed about the TSST protocol at the end of the assessment.

### 2.3. Measures

#### 2.3.1. Hemodynamic Measures

Data of systolic (SBP) and diastolic blood pressure (DBP) and heart rate (HR) were obtained continuously via a non-invasive hemodynamic monitoring system (Finapres^®^ Nova; Finapres Medical System, Enschende, The Netherlands) [12]. Markers for BP and HR were set under resting conditions 1 min before the start of the TSST procedure (Finapres^®^ Nova baseline reading) and immediately after stress as well as 15, 45 and 90 min after stress by Finapres^®^ Nova.

#### 2.3.2. Autonomic Measures

HRV was assessed with the Finapres^®^ Nova from continuous electrocardiogram (ECG) recordings. The Finapres^®^ Nova records the beat-to-beat finger pulse contour and assesses short-term changes of BP and its variability. A filter was applied to detect and remove abnormal beats. The root mean square successive difference (RMSSD) in heart period series was determined with Autonomic Testing software of the Finapres^®^ Nova. RMSSD is widely used in psychophysiological research as a measure of vagal cardiac influence [13]. RMSSD is more robust than other HRV indices because it is less influenced by breathing patterns [14]. RMSSD was calculated for each time point from 2-min segments of continuous data, which is acceptable to provide reliable estimates that correlate highly with 5-min recordings [14,15,16]. For the measurement of plasma norepinephrine and epinephrine levels, blood was drawn into EDTA-coated monovettes (ethylenediaminetetraacetic acid; Sarstedt, Numbrecht, Germany), and immediately centrifuged for 10 min at 2000× *g*. Obtained plasma was stored at −80 °C until biochemical analysis. Plasma norepinephrine and epinephrine were determined by high-pressure liquid chromatography (HPLC; detection limit: 0.25 pg/mL; inter- and intra-assay variance <5%; Laboratory for Stress Monitoring, Göttingen, Germany [17]). All stress hormone samples were determined from single measurements and all samples from each subject were analyzed in the same run to reduce systematic measurement errors. 

#### 2.3.3. Salivary Cortisol

For the assessment of salivary free cortisol levels, saliva was collected using Salivette collection devices (Sarstedt, Rommelsdorf, Germany) and stored at −20 °C until analysis. Thawed saliva samples were centrifuged at 3000 g for 10 min, yielding low-viscosity saliva. Salivary free cortisol concentrations were determined using a commercial chemiluminescence immunoassay with high sensitivity of 0.16 ng/mL (LIA) (IBL Hamburg, Germany). Intra- and inter-assay variability were <7.7% and 11.5%, respectively.

## 3. Results

### 3.1. Participants Characteristics

Table 1 depicts demographic, medical, and psychological characteristics of the monozygotic twins studied. Both show similarities in all characteristics. The TTS participant took her antihypertensive medication (Carvedilol) at home before the assessment.

### 3.2. Hemodynamic Measures 

**Figure 1:**
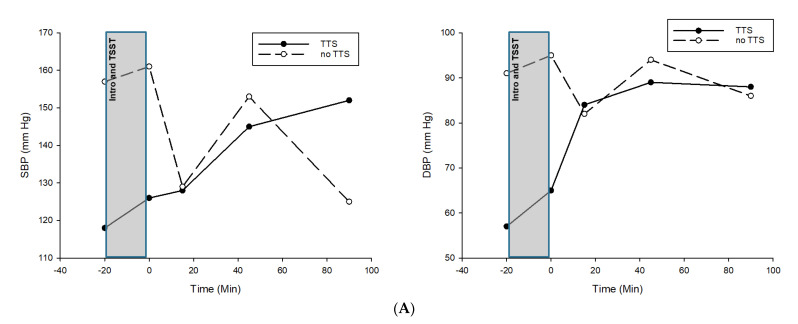

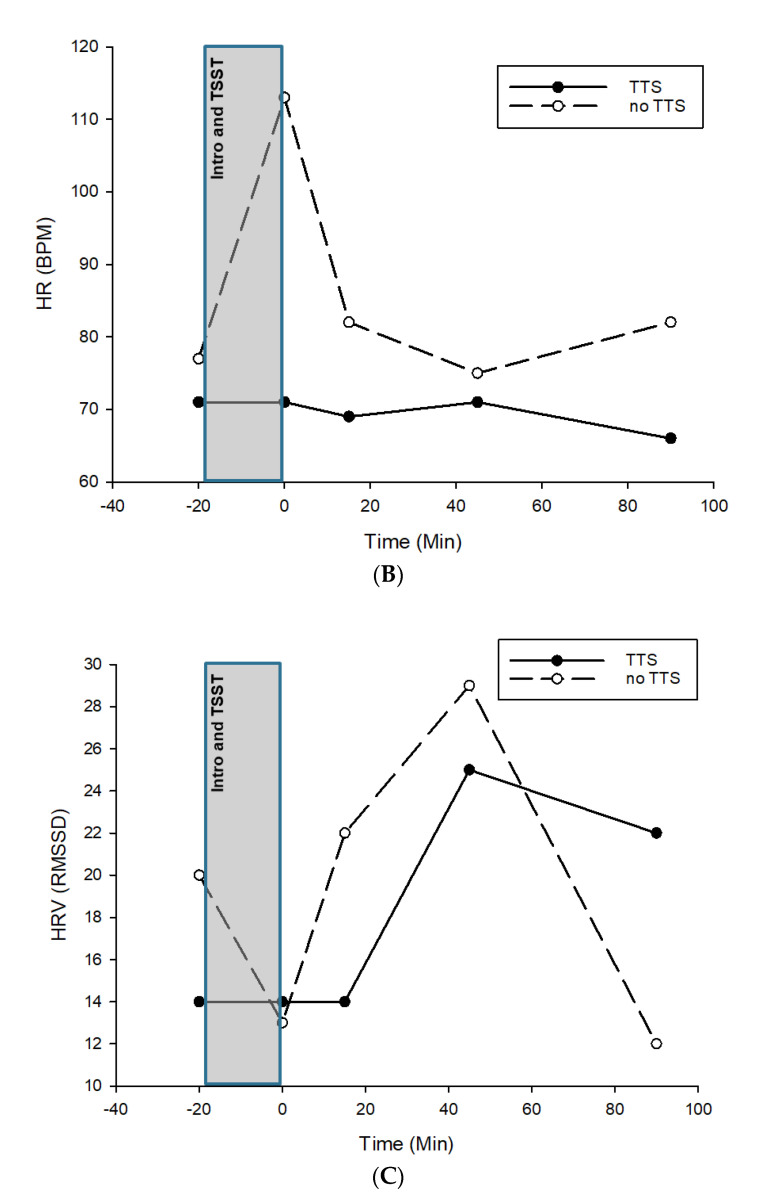
(**A**) Systolic blood pressure reactivity to TSST in TTS and non−TTS twin. Note: SBP = systolic blood pressure, DBP = diastolic blood pressure, TSST = Trier Social Stress Test, TTS = twin with Takotsubo syndrome, no TTS = twin with no Takotsubo syndrome. (**B**) Heart Rate reactivity to TSST in TTS and non-TTS twin. Note: HR = heart rate, BPM = beats per minute, TSST = Trier Social Stress Test, TTS = twin with Takotsubo syndrome, no TTS = twin no Takotsubo syndrome. (**C**) Heart Rate Variability reactivity to TSST in TTS and non-TTS twin. Note: HRV = heart rate variability, TSST = Trier Social Stress Test, TTS = twin with Takotsubo syndrome, no TTS = twin with no Takotsubo syndrome.

At baseline, SBP, DBP and HR were lower in the TTS twin compared to her non-TTS twin-sister. Likewise, the monozygotic twins differed in their hemodynamic stress response with the TTS twin showing blunted BP (Figure 1A), and HR (Figure 1B) reactivity. 

### 3.3. Autonomic Measures and Stress Hormones

Baseline RMSSD was somewhat lower in the TTS twin than in the non-TTS twin. Immediately after stress (+15 min) (Figure C) the TTS twin showed a lower absolute value in HRV. However, during the post-stress recovery period, RMSSD of the TTS twin increased implying higher HRV at 90 min after stress. 

**Figure 2:**
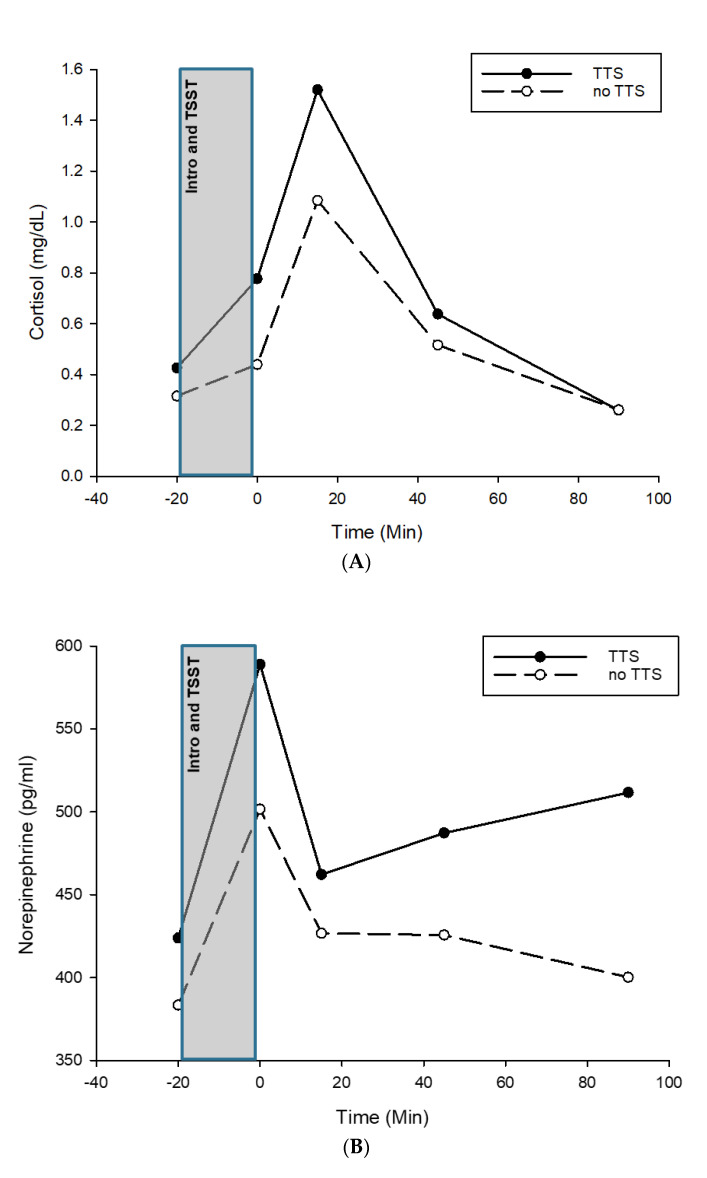

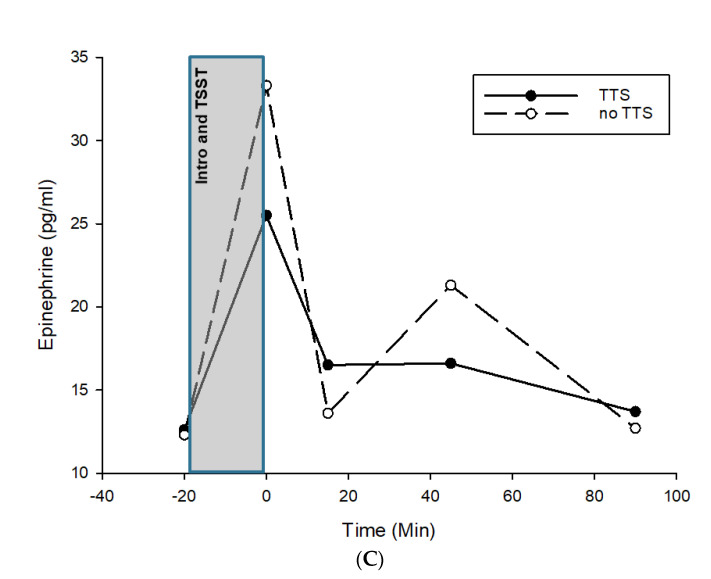
(**A**) Cortisol reactivity to TSST in TTS and non−TTS twin. Note: TSST = Trier Social Stress Test, TTS = twin with Takotsubo syndrome, no TTS = twin with no Takotsubo syndrome. (**B**) Norepinephrine reactivity to TSST in TTS and non-TTS twin. Note: TSST = Trier Social Stress Test, TTS = twin with Takotsubo syndrome, no TTS = twin with no Takotsubo syndrome. (**C**) Epinephrine reactivity to TSST in TTS and non-TTS twin. Note: TSST = Trier Social Stress Test, TTS = twin with Takotsubo syndrome, no TTS = twin with no Takotsubo syndrome.

The psychosocial stress test resulted in a cortisol, norepinephrine, and epinephrine response in both twins, with the TTS twin showing higher absolute values in all three hormones immediately after stress (+15 min) (Figure 2A–C). Whereas epinephrine levels immediately before stress were similar in both twins, baseline cortisol levels were somewhat higher and norepinephrine baseline levels markedly lower in the TTS twin compared with the non-TTS twin. While cortisol and epinephrine levels recovered to baseline in both participants 90 min after stress, this was the case for norepinephrine only in the non-TTS twin, while the TTS twin showed a steady increase in norepinephrine levels until 90 min after stress.

## 4. Discussion

In the present study, we found lower baseline values of BP and HRV in the TTS twin, whereas differences in absolute values of baseline HR, norepinephrine, epinephrine and salivary cortisol seemed to differ little if at all between the TTS twin and her non-TTS twin sister. However, in response to psychosocial stress, the TTS twin showed a relatively blunted reactivity in hemodynamic measures. This is contrary to the previous literature in which similar hemodynamic responses were reported for patients with TTS and healthy controls [7,8]. Moreover, we found lower RMSSD, compatible with greater vagal withdrawal immediately after stress, with a probably compensatory increase in vagal function during the recovery period from stress in the TTS twin. One previous study found unaltered HRV reactivity to acute stress, but it did not measure an index of pure vagal function [9]. Both catecholamines, norepinephrine more so than epinephrine, as well as cortisol showed greater stress reactivity in the TTS twin, with a further increase in norepinephrine release during the recovery period in the TTS twin. Whereas increased catecholamine reactivity has previously been shown in TTS [7,8], cortisol reactivity was unaltered in previous studies [7,9]. On the whole, these observations provide further evidence for altered autonomic reactivity and, as a novelty, also HPA reactivity in TTS.

There is an intriguing line of research that an excessive release of catecholamines critically contributes to myocardial stunning, a clinical hallmark of TTS. The reasons for the assumed vulnerability of the coronary microcirculation and cardiac myocytes to catecholamine effects is still largely unknown [6]. Our findings suggest that TTS patients may have a lower tolerance for physiological stress responses which may also occur more excessive. Large cohort studies are needed to confirm or dispute the findings of this case report and to particularly clarify the specific role of norepinephrine during psychosocial stress in patients with TTS. 

Apart from being a case report that does not allow conclusions based on formal statistical analysis, the use of carvedilol and an angiotensin-converting enzyme inhibitor, a common treatment regimen for patients after TTS, by the TTS twin is another limitation of our study. Due to ethical reasons, these medications were not discontinued for the stress test, but may have influenced stress responses. For instance, carvedilol is a lipophilic non-selective beta-blocker, which also selectively blocks alpha1-adrenoceptors. Non-selective beta-blockers have been shown to reduce HR reactivity to mild psychosocial stressors [18] and selective alpha1-adrenoreceptor blockade has been shown to reduce stress reactivity of systolic and diastolic BP [19]. Nevertheless, it is unlikely that the nearly absent response of HR and BP in the TTS twin was due to the medication alone. In contrast, nonselective beta-blockade did not previously influence the cortisol stress response differently from placebo in healthy subjects [20]. However, the additional effect of alpha1-blockade on cortisol reactivity or a specific effect of carvedilol on catecholamine and HRV reactivity has not be studied so far. Such effects could also be different in patients with TTS compared to healthy individuals.

In conclusion, our findings suggest that, in addition to genetic components, TTS is characterized by altered hemodynamic, autonomic and HPA-axis responses to acute psychosocial stress. Large patient registries employing a case-control design are needed to confirm blunted hemodynamic reactivity, increased catecholamine reactivity, vagal withdrawal, and increased cortisol reactivity in TTS. Careful matching of cases and controls in terms of cardiovascular medications that potentially influence physiological stress responses is critical to advance knowledge of pathophysiological mechanisms in TTS triggered by acute psychosocial stress.

## Figures and Tables

**Table 1:** Characteristics of the monozygotic twins with and without TTS.

	Takotsubo Twin	Non-TTS Twin
Age (years)	60	60
Gender	Female	Female
Civil status	Married	Married (2nd time)
Living status	With someone	With someone
Working status	Unemployed	Unemployed
Highest level of education	University (MSc in economics)	High school (nurse)
Children	2 (age: 25, 29)	2 (age: 28, 34)
Smoking	No (since age of 26)	No (since age of 40)
Alcohol consumption	1–2x/month	2x/month
Physical activity	No (active before TTS)	No
History of social phobia	Yes	Unclear
History of other anxiety disorders	Yes	Yes
Medication:		
Propranolol 20 mg	Yes (against anxiety disorder)	Yes (if required against anxiety disorder)
Estrogen	Yes	Yes
Progesterone	Yes	Yes
Angiotensin-converting enzyme inhibitor	Yes (since TTS)	No
Carvedilol	Yes (since TTS)	No

TTS = Takotsubo syndrome.

## Data Availability

All data used in this study are deposited and part of the University Hospital of Zurich, Switzerland.
